# Nonalcoholic fatty liver disease-related hepatocellular carcinoma growth rates and their clinical outcomes

**DOI:** 10.20517/2394-5079.2021.74

**Published:** 2021-11-05

**Authors:** Jihane N. Benhammou, Jonathan Lin, Elizabeth S. Aby, Daniela Markovic, Steven S. Raman, David S. Lu, Myron J. Tong

**Affiliations:** 1 Vatche and Tamar Manoukian Division of Digestive Diseases, David Geffen School of Medicine, Los Angeles, CA 90095, USA.; 2 Division of Gastroenterology, Hepatology and Parenteral Nutrition, Department of Medicine, VA Greater Los Angeles Healthcare System, Los Angeles, CA 90075, USA.; 3 Pfleger Liver Institute, University of California, Los Angeles, CA 90095, USA.; 4 Gastroenterology, Hepatology, Liver Transplantation and Nutrition, University of Minnesota, Minneapolis, MN 55455, USA.; 5 Department of Biomathematics, David Geffen School of Medicine at UCLA, Los Angeles, CA 90095, USA.; 6 Department of Radiology, David Geffen School of Medicine at UCLA, Los Angeles, CA 90095, USA.; 7 Liver Center, Huntington Medical Research Institutes, Pasadena, CA 91105, USA.

**Keywords:** Nonalcoholic fatty liver disease, hepatocellular carcinoma, tumor growth rates, biomarker

## Abstract

**Aim:**

Nonalcoholic fatty liver disease (NAFLD)-associated hepatocellular carcinoma (HCC) is projected to become the leading indication for liver transplantation. Previous studies indicate that tumor growth rates (TGR) may predict survival and were helpful in determining HCC surveillance intervals. Therefore, we aimed to determine its usefulness in predicting clinical outcomes and treatments.

**Methods:**

We conducted a retrospective study of hepatitis B, C and NAFLD-HCC cases. TGR was measured using 2-consecutive pre-treatment contrast-enhanced imaging studies ≥ 25 days apart. A multivariate regression model was used to determine predictors of TGR. In addition, the Cox regression model was used to evaluate the relationship between TGR and overall survival.

**Results:**

From 2000–2019, the study cohort comprised 38, 60, and 47 HBV, HCV, and NAFLD patients, respectively, with TGRs. NAFLD-HCC tumor size was inversely correlated to the extent of liver disease as measured by Child-Pugh score (7.2 cm in non-cirrhosis; 3.7 cm, 2.6 cm, and 2.1 cm in Child A, B, and C, respectively; *P* < 0.001). After adjusting for baseline characteristics, the TGR per month was fastest in HBV (9.4%, 95%CI: 6.3%-12.5%) compared to HCV (4.9%, 95%CI: 2.8%-7%) and NAFLD patients (3.6%, 95%CI: 1.6%-6.7%). Predictors of TGR included elevated AFP, low albumin, and smaller tumor size. Fast TGR in viral etiologies had higher mortality [adj. hazard ratio (HR) = 2.6, 95%CI: 1.2–5.7, *P* = 0.02] than slow TGRs, independent of treatments. Fast TGR in NAFLD had a trend towards higher mortality (HR = 3.6, 95%CI: 0.95–13.3, *P* = 0.059).

**Conclusion:**

NAFLD-HCC patients have more indolent growths than viral-related HCC TGRs. The addition of TGR as a biomarker may assist in stratifying treatment options.

## INTRODUCTION

Hepatocellular carcinoma (HCC) is the fourth leading cause of cancer-related death^[1]^. Although chronic hepatitis C (HCV) and B (HBV) continue to be the most common etiologies^[2]^, non-viral causes are increasing due to type 2 diabetes and the metabolic syndrome fueling the nonalcoholic fatty liver disease (NAFLD) epidemic worldwide^[3]^. NAFLD-related HCC is becoming the most common indication for liver transplantation in the United States, changing the landscape of HCC detection, surveillance, and management^[4]^. NAFLD patients also represent a distinct high-risk population due to the challenges of ultrasound-based HCC screening related to increased subcutaneous, visceral, and intrahepatic adiposity^[5]^; the potential absence of elevated tumor markers, such as alpha-fetoprotein (AFP)^[6]^; and the observation that 20%-30% of all HCCs occur in the absence of underlying cirrhosis^[7]^. Prior studies on HCC tumor growth rate (TGR) helped inform society guidelines for optimal bi-annual screening intervals for HCC surveillance^[8–10]^. This has improved HCC survival from early detection and early triage to curative-intent therapies such as surgery and ablation^[11,12]^. However, these strategies are based mainly on HBV patients before NAFLD was recognized as a disease, and thus further NAFLD-specific studies are required to optimize HCC management.

Recent TGR studies confirm heterogeneity in HCC, commonly seen in clinical practice^[13,14]^. However, despite current work assessing TGR, NAFLD patients continue to be under-represented. Therefore, given the advances in radiological technology over the years and the changing landscape of HCC, further work is needed to understand TGRs in the NAFLD population. Understanding how TGR differs from other etiologies of HCC and how these, in turn, affect overall survival and clinical outcomes is imperative to guide future management and surveillance practices. In the current study, we aimed to derive HCC TGR in a NAFLD sub-cohort and contemporaneously compare to HCC TGR in HBV and HCV sub-cohorts to determine how etiology-specific TGR impacted clinical outcomes, including overall survival based on treatment modalities.

## METHODS

The study was approved by the Institutional Review Board of the University of California, Los Angeles (UCLA) (IRB#17–000015) and was performed in compliance with the 1996 United States Health Information Portability and Accountability Act.

### Data source

This is a retrospective case-case comparison of consecutive NAFLD-associated HCC (including NASH) to contemporaneous HBV- and HCV-associated HCC cases. The NAFLD sub-cohort cases were derived using a combination of free natural language processing and the *International Classification of Diseases* (ICD-9 and ICD-10 codes), as previously described^[6]^. NAFLD and NASH cases were identified based on the review of histopathology^[15]^ and/or clinical assessment by a hepatologist^[6]^. HBV and HCV patients were identified from one high volume, outpatient liver disease referral clinic (Liver Cancer Center in Pasadena, CA). In addition, they included those subsequently referred to a high volume, tertiary academic transplant center (Dumont-UCLA Liver Cancer Center, Los Angeles, CA) for liver transplantation evaluation^[16]^. Thus, we identified 333 HBV and HCV cases. Four HBV and HCV patients were excluded due to having coinfections. Therefore, the study cohort comprised 467 patients with HCC, of which the NAFLD, HBV, and HCV sub-cohorts comprised 138, 170, and 159 patients, respectively.

All study cohort patients had a multiphasic contrast-enhanced computerized tomography (CT) or magnetic resonance imaging (MRI). In addition, all cases included in the study diagnosed with HCC were classified as LR-5 based on the Liver Imaging Reporting and Data System (LI-RADS-5)^[17]^ criteria on multiphasic (arterial, portal venous and delayed) contrast-enhanced CT or MRI or histologically confirmed on liver biopsy, resection, explant or autopsy (in the event of death).

### Baseline patient and HCC tumor characteristics

Patient demographics and laboratory data were collected at the time closest to HCC diagnosis. HCC number and size were obtained for all patients from imaging or pathology. We prioritized review of imaging studies from the tertiary academic center, followed by outside studies reinterpreted by academic abdominal radiologists, and finally outside studies if no local studies were available for review or reinterpretation. Abdominal ultrasound results were excluded.

HCC cases were classified using both the Milan criteria (single lesion 5 cm, maximum of three lesions with none > 3 cm) and also the University of California at San Francisco (UCSF) criteria (single lesion 6.5 cm, maximum of three lesions with none > 4.5 cm, or a total tumor burden of 8 cm). The presence of metastasis was determined based on CT or MRIs of the chest and abdomen and technetium 99m labeled bone scans.

### Tumor growth rate measurements

The HCC TGR was calculated from multiphasic contrast-enhanced CTs or MRIs of patients who had two consecutive studies prior to any HCC therapy, at least 25 days apart^[14]^. Of the initial study cohort of 467 patients with the three etiologies of liver disease, 191 had at least two consecutive CTs or MRIs for TGR measurement from 1973–2019. Among the 191, 145 patients (38 HBV, 60 HCV, and 47 NAFLD) had two consecutive imaging studies from 2000–2019 (cohort is presented in Supplementary Figure 1). All NAFLD patients except one were entered into the study from 2000–2019. All multiphasic contrast-enhanced CT and MRIs, including the HBV and HCV cases initially evaluated at the outpatient liver clinic, were re-analyzed by experienced liver radiologists (Lu DS or Raman SS) at the liver transplant center starting in the year 2000. Therefore, the final cohort used for TGR measurements comprised of consecutive scans from 2000–2019. If patients had > 1 LI-RADS 5 lesion, the largest lesion was used for tumor measurement. Given that the HBV and HCV cases only included patients with a background of cirrhosis, non-cirrhosis NAFLD cases were excluded from the TGR measurements and comparisons.

### Clinical outcomes

We determined the treatment types throughout their clinical course for each patient until the last day of follow-up through Feb 20th, 2020, or death. Given that many patients would receive locoregional therapy as a bridge to liver transplantation, we classified most to least definitive treatment as follows: orthotopic liver transplantation, surgical resection, radiofrequency ablation, trans-arterial chemoembolization/Y-90, chemotherapy, based on prior work^[6,16]^. Therefore, for clinical outcomes, only the most definitive therapy was used in the final analyses.

### Statistical analysis

Categorical variables were compared by diagnosis using the Chi-square/Fisher’s test as appropriate. Continuous variables were compared across the groups using the Wilcoxon rank-sum test.

#### Tumor growth rate

The data were subdivided into all eras (1973–2019) and the most recent era (2000–219) to adjust for time variable and radiologist inter-observer differences.

TGR was computed as % per month growth using the Schwartz formula^[1]^ for tumor doubling time (TDT):

$$TDT=(T-T_{0})\;\ln2/[\ln(V/V_{0})]$$

TDT is directly related to TGR by the following formula:

$$\text{TGR}=\ln2/TDT=\ln(V/V_{0})/(T-T_{0})$$

TGR was therefore estimated assuming an exponential growth rate model. This model shows that tumor size on the log scale is linearly related to the time variable corresponding with the above formula. Next, the percent TGR was compared across diagnoses using the Wilcoxon rank-sum test. Finally, the percent TGR was compared across diagnoses using a multivariable quantile (median) regression model for the adjusted analysis after controlling for potential confounders including age, sex, log AFP, platelets, surveillance, Child-Pugh score, diabetes, and initial tumor size. The final model was selected using the backward procedure for variable selection and *P* < 0.15 as the retention criterion.

#### Predictors of tumor growth rate

To assess which co-variables were predictive of higher TGRs, we used a multivariable classification and regression tree model^[18]^ based on the following candidate predictors: age, sex, AFP, albumin, Child-Pugh score, diabetes, surveillance, era, and initial tumor size. All 191 HCC patients (1973–2000) were included to increase the statistical power of our final cohort for the tree model analysis. The regression tree method looks at every value of each predictor variable and temporarily splits the dataset into a non-overlapping “right” node subset and a “left” node subset. It then determines how “different” the cases in the two nodes are from each other (at least on average) and/or how similar all the cases inside the same node are to each other (the within node “purity” or “homogeneity”) based on the outcome measure. After going through every possible value of every predictor variable, the dataset is split permanently using that variable value which creates the greatest between node differences and/or the greatest within node “purity”. This process is then independently repeated in the two “daughter” nodes created by the split. Splitting continues until the data are too sparse (*n* < 5) or the difference between the two nodes is not statistically significant (*P* < 0.15). The best split for this model was determined by the impurity criterion, a reduction of the residual sum of squares due to the binary split (GINI criterion^[19]^).

#### Survival analyses

A Cox proportional hazard model was used to assess the association between TGR predicted categories (low, medium, fast) *vs*. mortality separately by diagnosis after adjusting for covariates. The Hazard ratio (HR) and its 95% confidence bound are reported. Overall survival curves were constructed for each TGR category using the Kaplan-Meier method separately by diagnosis and treatment type. Finally, survival curves were compared across the TGR categories using the log-rank test.

## RESULTS

### Patient and tumor characteristics

TGR analysis was performed on the final study cohort of 145 consecutive patients (38 HBV, 60 HCV, and 47 NAFLD) with two multiphasic CT or MR studies obtained between 2000 and 2019 and re-reviewed expert abdominal radiologists. Men comprised the majority of the HBV (*n* = 29, 76%) and HCV (*n* = 40, 67%) sub-cohorts and a minority of the NAFLD sub-cohort (*n* = 19, 40%) (*P* = 0.00016) [Table 1]. Although the NAFLD patients had more women compared to the viral etiologies, there were no differences in the clinical presentations of men and women except serum AFP level at the time of HCC diagnosis, where women were more likely to have a higher AFP with a median of 9 ng/mL (IQR: 4.9–45.0), compared to men who presented with a median of 4 ng/mL (IQR: 3–6.7, *P* = 0.016) [Supplementary Table 1]. Patients in the HBV sub-cohort were younger than patients in the HCV and NAFLD sub-cohorts (62 *vs*. 67 and 64 years, respectively; *P* = 0.003), while patients in the NAFLD HCC sub-cohort were more likely to have type 2 diabetes compared to those in the HBV and HCV sub-cohorts (*n* = 35, 74%; *P* < 0.0001). Within the viral sub-groups, 52 patients with HBV (31%) were on antiviral therapy at the time of HCC diagnosis, while 15 patients with HCV (9%) had reached sustained virologic response (SVR) at the time of HCC diagnosis. Patient in the NAFLD sub-cohort were more likely to present with stigmata of decompensated liver disease at HCC diagnosis including hepatic encephalopathy [NAFLD (23%), HBV (1%) and HCV (5%); *P* = 0.0017], ascites [NAFLD (34%), HBV (0%), HCV (6%); *P* < 0.0001] and Child-Pugh (CP) category B [NAFLD (41%), HBV (5%) and HCV (13%); *P* = 0.0004] compared to A [NAFLD (52%), HBV (95%) and HCV (85%); *P* = 0.0004]. Although NAFLD patients were more likely to have features of the metabolic syndrome [Table 1], a minority (*n* = 29, 21%) were on statin therapy at the time of HCC diagnosis when referred for further management.

Patients in the NAFLD sub-cohort were also less likely to undergo HCC surveillance (60%) compared to the patient in the HBV (74%) and HCV (82%) sub-cohorts (*P* = 0.039). Patients in the NAFLD, HBV, and HCV sub-cohorts were likely to have HCC tumors within Milan and UCSF criteria [Table 2]. Patients with HCC in the NAFLD sub-cohort were more likely to receive liver transplantation as definitive therapy (43%) compared to patients in the HBV (16%) or HCV (22%) sub-cohorts (*P* = 0.0146).

### Tumor growth rates

For TGR measurements, the median time between two consecutive imaging studies for HBV, HCV, and NAFLD sub-cohorts was 3 months (IQR: 2.2–16.2 months), 4.1 months (IQR: 1.9–19.4 months), and 3.1 months (IQR: 1.9–5.3 months) (*P* = 0.200), respectively. Within the NAFLD sub-cohort, 17% (24/138) of patients did not have underlying cirrhosis as defined by fibrosis stage on pathology (biopsy, resection, or explant tissues) or clinical diagnosis by a hepatologist^[6]^. The mean HCC size for patients without underlying cirrhosis was larger (7.2 cm, SD ± 4.7 cm) than patients with cirrhosis [3.2 cm, SD ± 2.5 cm, (*P* < 0.001)]. Patients with underlying CP-A (*n* = 47) liver disease had a larger mean tumor size at presentation [3.7 cm, SD ± 2.8 cm (*n* = 39)] compared to CP-B [2.6 cm, SD ± 1.3 (*n* = 39)] and CP-C [2.1 cm, SD ± 0.7 cm (*n* = 9)] (*P* < 0.001) without significant differences between CP-B and CP-C subgroups (*P* = 0.274).

Given differences in tumor size based on the cirrhosis status of NAFLD patients and that HBV and HCV sub-cohorts only comprised of patients with cirrhosis, NAFLD patients without cirrhosis were excluded in the TGR analyses. In the unadjusted model, the median HCC TGR for HBV, HCV and NAFLD sub-cohorts were 6.2% (IQR: 2.6%-12.2%), 5.5% (IQR: 1.2%-11.5%) and 3.8% per month (IQR: 0.0–11.3%, *P* = 0.48) respectively. We did not identify any differences in % TGR between men (median 5%, IQR: 2%-13%) and women (median 4%, IQR: 0–11%, *P* = 0.558). There were no significant differences in HCC TGR as a function of initial tumor size for all three HCC etiologies [Supplementary Table 2]. Of note, no differences in % TGR were observed in NAFLD sub-cohort population, with men presenting with a median of 5% per month (IQR: 2%-13%) and women with a median of 4% per month (IQR: 4%-11%, *P* = 0.558).

**Figure 1.**
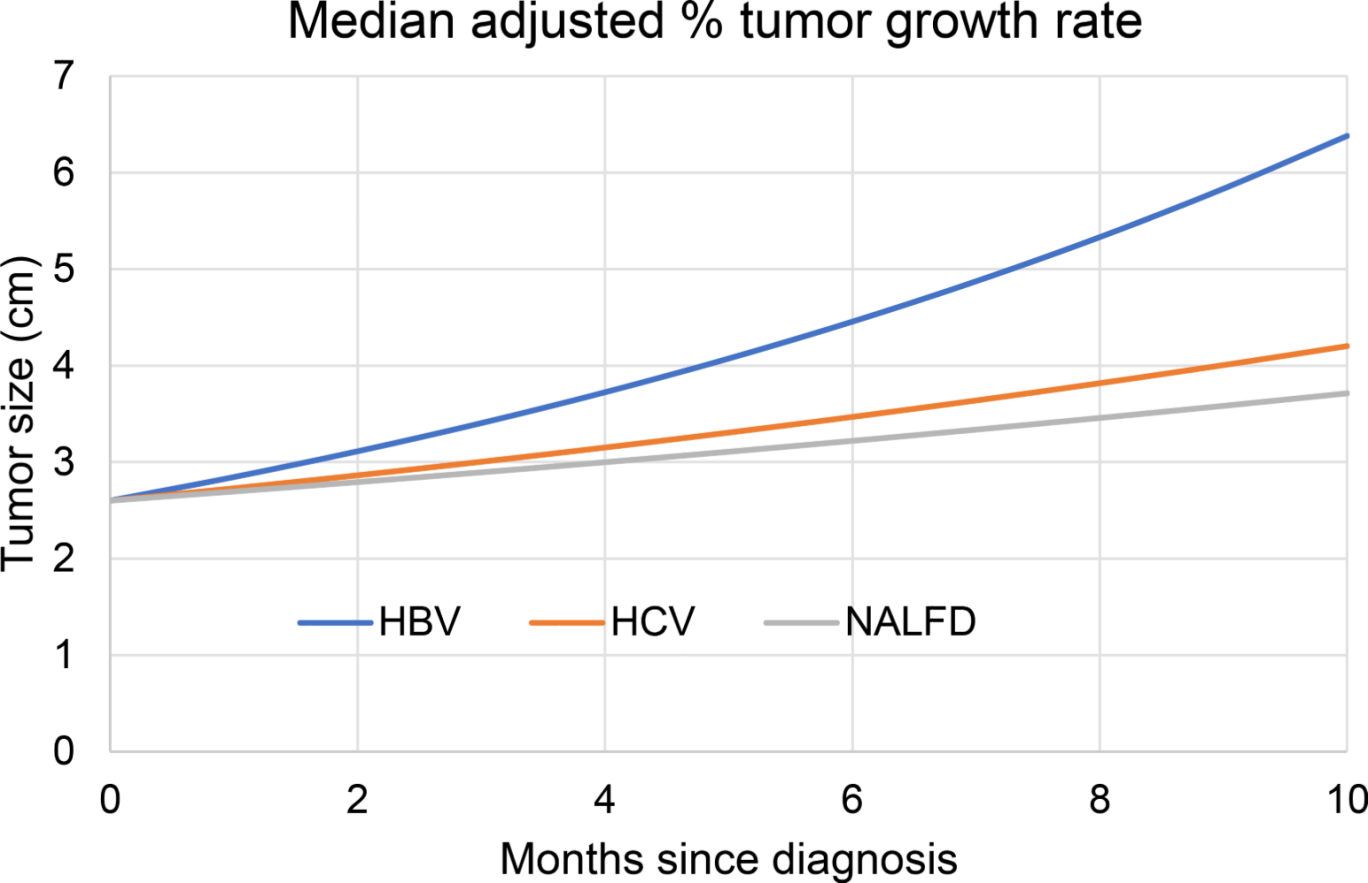
Median adjusted (adjusted for AFP, albumin, and initial tumor size) HCC tumor growth rates (TGR) in HBV, HCV, and NAFLD (*n* = 145). HCC: Hepatocellular carcinoma; HCV: hepatitis C virus; HBV: hepatitis B virus; NAFLD: nonalcoholic fatty liver disease; AFP: alpha-fetoprotein.

Next, we evaluated the relationship between diagnosis and HCC TGR in a multivariable quantile regression analysis to adjust for potential confounders. Among age, sex, AFP, albumin, CP score, receipt of surveillance, platelets, total bilirubin, type 2 diabetes, and initial tumor size, we found that higher AFP (*P* = 0.048), lower albumin at presentation (*P* = 0.028), and lower initial tumor size (*P* = 0.025) were associated with higher TGR. Other potential confounders tested were not associated with HCC TGR, including platelet count (*P* = 0.971) and total bilirubin (*P* = 0.752) at the time of presentation. In the adjusted multivariable model that included those co-variables, HBV sub-cohort patients had a higher TGR at 9.4% per month (95%CI: 6.3%-12.5%) compared to HCV and NAFLD sub-cohort patients 4.9% TGR (95%CI: 2.8%-7%) and 3.6% (95%CI: 1.6%-6.7%), respectively [Figure 1]. In pairwise HCC TGR comparisons, the HBV sub-cohort patients had higher HCC TGR than NAFLD (*P* = 0.014). No HCC TGR differences were detected between HCV and HBV sub-cohort patients (*P* = 0.061) or NAFLD and HCV sub-cohort patients (*P* = 0.525).

### Predictors of TGR

**Figure 2.**
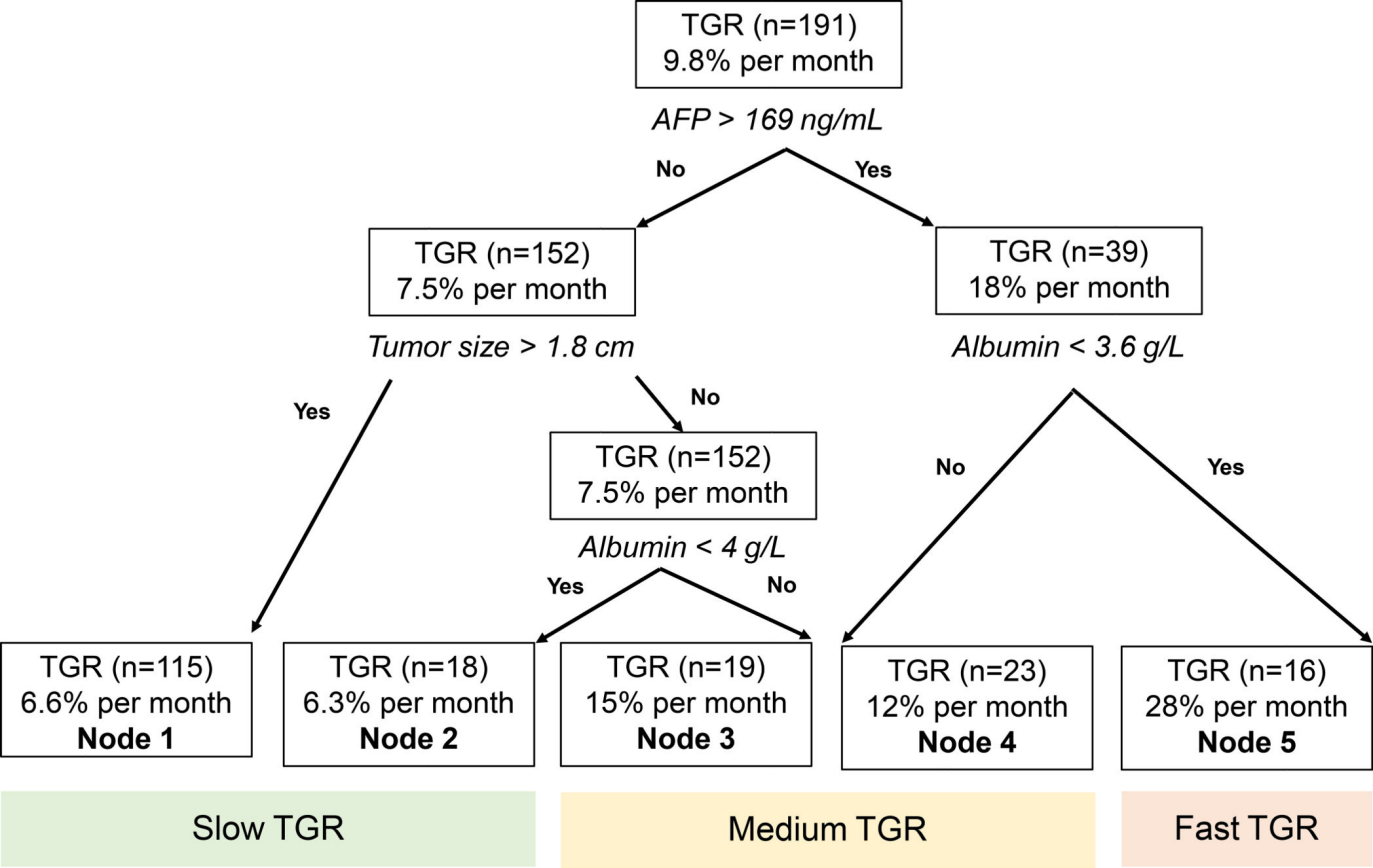
Regression tree model for predicting HCC % TGR per month for all etiologies of HCC (*n* = 191). TGR: Tumor growth rates; AFP: alpha-fetoprotein; HCC: hepatocellular carcinoma.

To further stratify our data into key clinical parameters that affected HCC TGR, we developed a tree model of the predictors that included age, sex, era, AFP, albumin, CP score, receipt of surveillance, and initial tumor size in the entire 191 HCC TGR cohort. Of those other predictors, we identified that AFP of > 169 ng/mL followed by an initial tumor size of 1.8 cm and an albumin threshold of 3.6 g/dL were the best discriminators of “slow” *vs*. “fast” TGR. Our tree model identified 5 nodes [Figure 2] that sub-classified the TGR by AFP, initial tumor size, and albumin. To better understand how HCC TGR affected clinical outcomes for each HCC etiology, we classified nodes 1 (*n* = 115) and 2 (*n* = 18) as having “slow” TGR (6.6% and 6.3% per month, respectively). Nodes 3 (*n* = 19) and 4 (*n* = 23) were considered to have “medium” TGR (15% and 12% per month, respectively) and node 5 (*n* = 16) had the “fast” TGR at 28% per month [Figure 2]. In the sub-set of patients evaluated from 2000–2019 (*n* = 145), we found similar associations between TGR and overall mortality based on etiology and treatment modality [Supplementary Table 3].

### HCC TGR and overall survival

**Figure 3.**
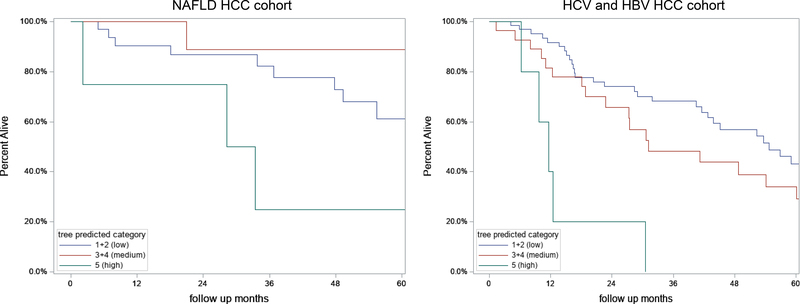
Kaplan Meier Survival by TGR tree predicted categories stratified by etiology in the 191 patients with HCC. (A) NAFLD HCC cohort; (B) HBV and HCV HCC cohorts combined. HCC: Hepatocellular carcinoma; HCV: hepatitis C virus; HBV: hepatitis B virus; NAFLD: nonalcoholic fatty liver disease.

We then determined the association between TGR and overall survival in the three-node growth patterns for the NAFLD, HBV, and HCV sub-cohorts [Figure 3]. In the unadjusted model for the NAFLD sub-cohort (*n* = 47), HCC tumors with fast TGR (*n* = 4) had higher mortality (HR = 3.6, 95%CI: 0.95–13.3, *P* = 0.059) compared to HCC tumors with slow TGR (*n* = 33). We combined HBV and HCV sub-cohorts into a viral sub-cohort to determine overall survival since there was no significant difference in HCC TGR between the HBV and HCV sub-cohorts. In the unadjusted model, HBV and HCV patients with fast HCC TGR (*n* = 12) had significantly higher overall mortality (HR = 2.5, 95%CI: 1.3–4.8, *P* = 0.005), compared to patients with slow HCC TGR (*n* = 100). Of note, there was no significant overall survival difference between men (63.3%) and women (62.8%) in the NAFLD sub-cohort (*P* = 0.789). After adjusting for baseline characteristics (age, sex, CP score, AFP, and surveillance), patients with fast HCC TGR in the overall study cohort significantly increased overall mortality compared to patients with slow HCC TGR (adj. HR = 2.6, 95%CI: 1.2–5.7, *P* = 0.02).

#### TGR and overall survival based on most definitive treatment

We next stratified our results based on the most definitive treatment modality received by the patient for all etiologies of HCC. In patients undergoing liver transplantation, fast HCC TGR patients (*n* = 4) had a significantly increased risk of overall mortality compared to slow HCC TGR patients (*n* = 28) (HR = 4.1, 95%CI: 1.1–15.7, *P* = 0.04). Fast HCC TGR patients (*n* = 4) had significantly increased overall mortality (HR = 5.4, 95%CI: 1.8–16.0, *P* = 0.002). After adjusting for covariates, we found that fast HCC TGR patients had an increased risk of overall mortality (adj. HR = 6.6, 95%CI: 1.1–38.6, *P* = 0.04).

## DISCUSSION

The etiology of HCC is rapidly changing in the United States with the increase in metabolic syndrome and its liver sequela, NAFLD. Previous studies of HCC arising from viral etiologies provided a framework for imaging surveillance intervals and treatment strategies in those etiologies. However, to optimize care, tumor heterogeneity and the clinical and biological differences that drive HCC development should be reevaluated in the era of NAFLD-derived HCC. This study found significant HCC TGR differences in the largest NAFLD population to date compared to HBV and HCV etiologies before and after definitive treatment.

First, NAFLD sub-cohort patients were more likely to present with decompensated liver disease at the time of HCC diagnoses compared to HBV and HCV sub-cohorts. Second, within the NAFLD sub-cohort, there was an inverse relationship between the extent of underlying liver disease and tumor size with non-cirrhosis patients presenting with the largest tumors. Third, HBV sub-cohort patients had significantly faster HCC TGR compared to the NAFLD sub-cohort. Fourth, AFP, albumin, and initial tumor size independently predicted HCC TGR. Finally, fast HCC TGR was associated with increased overall mortality, independently of the most definite HCC treatment.

### Tumor size differences are identified in NAFLD-related HCC

We found that NAFLD patients without cirrhosis were more likely to present with HCC tumors exceeding Milan and UCSF criteria compared to NAFLD patients with cirrhosis. Although this may partly reflect of lack of HCC surveillance in the non-cirrhosis population, differences in underlying hepatocarcinogenetic mechanisms are also likely. The size differential based on the extent of underlying liver disease, as defined by CP score, suggests that the lipid-rich and/or pro-inflammatory environment may trigger HCC development independently of the cycle of inflammation and attempted healing in fibrotic pathways^[20]^. We also noted that, unlike HBV and HCV patients, NAFLD patients were more likely to present with advanced liver disease and were more likely to undergo liver transplantation as a definitive treatment. This could be partly driven by the lack of population screening for NAFLD, delaying its detection. Although the incidence of NAFLD-derived HCC remains lower than viral etiologies, the epidemic proportion of patients estimated to live with NAFLD will continue to increase NAFLD-related HCC cases worldwide proportionally^[21]^. Thus, there is a significant unmet need to develop clinical risk assessment tools and biomarkers to screen NAFLD patients at risk for developing HCC. A recent study by Noureddin *et al*.^[22]^ showed that screening for NAFLD within the type 2 diabetes population using non-invasive methods is cost-effective using a Markov model. Early detection of high-risk NAFLD patients, such as ones with type 2 diabetes, may prove to be an effective strategy to intervene early, preclude liver decompensation from occurring and offer a range of potential curative-intent therapeutic options in addition to liver transplantation as with patients with viral etiologies of HCC.

### Viral etiologies of HCC have faster TGR compared to NAFLD-related HCC

Our comparative studies revealed that HBV sub-cohort patients had more rapid HCC TGRs than HCV and NAFLD sub-cohort patients in line with prior studies reporting a variance in HCC TGRs based on chronic liver disease etiology^[13,23,24]^. Rich *et al*.^[13]^ studied HCC TGR patterns in a heterogeneous group of patients in western countries and reported that non-viral HCC etiologies had more indolent growth than viral causes. However, 70% of their study cohort comprised HCV patients (*n* = 169) while only 6.6% and 11.1% were comprised of NAFLD (*n* = 16) and HBV (*n* = 8) patients, respectively. The effect of TGR on overall survival was preliminarily assessed in a subset of their cohort (*n* = 184), demonstrating that indolent HCC tumors from mostly non-viral HCCs, had improved overall survival (adj. HR = 0.61, 95%CI: 0.40–0.95) before adjusting for treatments. Our detailed pre-treatment TGR analyses in the different “most definitive” treatment modalities explore these associations further and demonstrate that TGR affects overall survival in surgical and locoregional therapy groups. These associations suggest that treatment algorithms should be developed beyond the currently used tumor burden, extra-hepatic disease, and AFP biomarker. We also noted that a minority of patients with HCV HCC had reached SVR (9%) at the time of HCC diagnosis. While the risk of HCC has been shown to decrease post SVR, it is not eliminated^[25]^. In the era of direct-acting antiviral therapy, understanding the natural history of HCC TGR in the post-SVR patient population will need to be re-assessed. This will be especially crucial in HCV patients who have reached SVR with concomitant metabolic syndrome risk factors, which has been shown to increase the risk of HCC development^[26]^.

### TGR is a potential biomarker for HCC treatment decisions

HCC therapies have significantly evolved. Deciding on the type of HCC treatment is complex and requires a multidisciplinary approach to assess the disease burden, patient functional status, and liver dysfunction. Although treatment selection has primarily been based on the number and size of HCC tumors (UCSF and Milan criteria) and AFP level^[27]^, biomarkers such as cell-free DNA and methylation technologies have also have been investigated^[28]^. Response to locoregional treatment has proven to be an effective biomarker to determine tumor biology, which has led to change in HCC transplant allocation policies by the United Network of Organ Sharing^[29,30]^. Our data suggest that HCC TGR based on the step-wise tree model affects overall survivals of patients and should be considered a biomarker in treatment selection.

### Limitations

While we present the largest comparative analysis of NAFLD and non-NAFLD HCC TGR to our knowledge, our study has limitations. The sample size for sub-cohort analyses is likely underpowered to detect subtle differences among sub-cohorts, and larger case-control, prospective multi-institutional studies are required. Our findings identify important associations between TGR and survival based on etiology, which must be validated. As most patients evaluated at the tertiary hospital and clinic were largely referred for their HCC management, granular data on the cause of death is lacking, making the assessment of cancer-specific death not feasible in our study. In addition, residual confounding is possible given the retrospective nature of the study. We did not adjust for known risk factors for poor HCC outcomes after treatment, including tumor differentiation and microvascular invasion. However, these data are not readily available in real-world practice given the lack of tissue sampling prior to diagnosis and therapy. Our data also demonstrate that some patients had delays in surveillance imaging, which is likely reflective of limitations seen in clinical practice due to systems and patient-related barriers in diagnosis and treatment initiation^[31,32]^. Although statin therapy was reported in the NAFLD cohort, statin use in the HBV and HCV cohorts was not available. Similarly, the use of aspirin was also not available for any sub-cohort. As lipophilic statins and aspirin have been associated with improved HCC survivals in large epidemiological studies^[33,34]^, adjusting for their use in multivariable models and/or determine how their use affects TGR will be important in future work.

### Future directions

Our study has several key clinical implications. First, although previous work on viral etiologies of HCC has been instrumental in guiding practices, the changing landscape of HCC due to NAFLD prompts reassessing previous work that has shaped the current management of our patients. Second, as more work is being conducted on identifying biomarkers to understand HCC biology and define treatment algorithms, the use of TGR should be considered. Third, in the era of improved outcomes with locoregional therapy and the advent of HCC immunotherapy, early understanding of TGR trajectory by measuring 2-consecutive cross-sectional images to guide therapy may refine our treatment strategies, especially when considering liver transplantation, which remains a scare resource. Finally, extensive, multicenter, prospectively collected data more inclusive of NAFLD patients will be needed to provide patient-centered care.

## Supplementary Material

Supplemental figure 1

Supplemental tables 1-3

## Figures and Tables

**Table 1.** Baseline patients characteristics (*n* = 145)

	HBV (38)	HCV (60)	NAFLD (47)	*P* value
Males *n*, %	29 (76)	40 (67)	19 (40)	0.0016
Women n, %	9 (24)	20 (33)	28 (60)	
Mean age at HCC dx ± SD	62 ± 11	67 ± 10	64 ± 7	0.0030
Type 2 diabetes, *n* (%)	8 (21)	17 (29)	35 (74)	< 0.0001
Decompensation, *n* (%)				
*HE*	1 (3)	3 (5)	11 (23)	0.0017
*Ascites/volume overload*	0 (0)	2 (6)	19 (34)	< 0.0001
Child-Pugh score				
*A*	36 (95)	51 (85)	23 (52)	< 0.001
*B*	2 (5)	8 (13)	18 (41)	
*C*	0 (0)	1 (2)	3 (7)	
Median INR (IQR)	1.1 (1.0–1.2)	1.1 (1.1–1.2)	1.2 (1.1–1.3)	0.0530
Median AST (IQR)	50 (25–52)	85 (44–114)	49 (35–60)	< 0.0001
Median ALT (IQR)	39 (26–59)	58 (35–107)	32 (23–39)	< 0.0001
Median albumin (IQR)	4.2 (3.9–4.7)	3.8 (3.4–4.2)	3.6 (3.2–4.0)	< 0.0001
Median total bilirubin (IQR)	0.8 (0.5–0.9)	1.1 (0.8–1.6)	1.3 (0.8–2.3)	0.0010
Median platelets (IQR)	170 (95–195)	115 (77–186)	94 (65–146)	0.0010
Screened for HCC, *n* (%)	28 (74)	49 (82)	28 (60)	0.0391
Family history HCC, *n* (%)	14 (39)	1 (2)	2 (4)	< 0.0001
Family history LD, (%)	4 (29)	6 (11)	16 (34)	0.0130

HCC: Hepatocellular carcinoma; INR: international normalized ratio; AST: aspartate aminotransferase; ALT: alanine aminotransferase; LD: liver disease.

**Table 2.** Tumor characteristics and treatments in the different etiologies of HCC (*n* = 145)

	HBV (38)	HCV (60)	NAFLD (47)	*P* value
Within Milan (%)	31 (82)	51 (85)	42 (89)	0.592
Within UCSF (%)	31 (82)	57 (95)	44 (94)	0.060
Median first tumor size (cm) (IQR)	2.8 (1.6–4.3)	2.5 (1.9–3.6)	2.3 (1.7–3.2)	0.619
Median AFP at presentation (IQR)	7.7 (3.7–124)	32.8 (11.2–149)	6.1 (3.9–15)	< 0.0001
Most definitive therapy, *n* (%)				
*OLT*	6 (16)	13 (22)	20 (42)	0.0146
*Resection*	10 (26)	6 (10)	1 (2)	
*RFA*	9 (24)	15 (25)	14 (30)	
*TACE/Y-90*	7 (18)	9 (15)	2 (4)	
*PEI*	0 (0)	1 (2)	0 (0)	
*Chemotherapy*	3 (8)	3 (5)	3 (6)	
*Supportive*	3 (8)	13 (22)	5 (11)	
Previous treatment prior to most definitive, *n* (%)				
*RFA prior to OLT*	4 (11)	9 (15)	19 (40)	-
*TACE prior to OLT*	8 (21)	9 (15)	16 (34)	
*Others*	5 (14)	7 (12)	7 (15)	

IQR: Interquartile range; AFP: alpha-fetoprotein; RFA: radiofrequency ablation; TACE: trans-arterial chemoembolization; OLT: orthotopic liver transplantation.

## References

[1] Schwartz M A biomathematical approach to clinical tumor growth Cancer 1961;14:1272–94. 13909709 10.1002/1097-0142(196111/12)14:6<1272::aid-cncr2820140618>3.0.co;2-h

[2] Yang JD, Roberts LR. Hepatocellular carcinoma: a global view. Nat Rev Gastroenterol Hepatol 2010:7:448–58. 20628345 10.1038/nrgastro.2010.100 PMC3926946

[3] Estes C, Razavi H, Loomba R, Younossi Z, Sanyal AJ. Modeling the epidemic of nonalcoholic fatty liver disease demonstrates an exponential increase in burden of disease. Hepatology 2018;67:123–33. 28802062 10.1002/hep.29466 PMC5767767

[4] Younossi ZM, Otgonsuren M, Henry L, Association of nonalcoholic fatty liver disease (NAFLD) with hepatocellular carcinoma (HCC) in the United States from 2004 to 2009. Hepatology 2015;62:1723–30. 26274335 10.1002/hep.28123

[5] Simmons O, Fetzer DT, Yokoo T, Predictors of adequate ultrasound quality for hepatocellular carcinoma surveillance in patients with cirrhosis. Aliment Pharmacol Ther 2017;45:169–77. 27862091 10.1111/apt.13841 PMC7207219

[6] Benharnmou JN, Aby ES, Shitvanian G, Manansala K, Hussain SK, Tong MJ. Improved survival after treatments of patients with nonalcoholic fatty liver disease associated hepatocellular carcinoma. Sci Rep 2020; 10:9902. 32555268 10.1038/s41598-020-66507-7 PMC7303220

[7] Kanwal F, Kramer JR, Li L, Effect of metabolic traits on the risk of cirrhosis and hepatocellular cancer in nonalcoholic fatty liverdisease. Hepatology 2020;71:808–19. 31675427 10.1002/hep.31014

[8] Barbara L, Berrzi G, Gaiarri S, Natural history of small untreated hepatocellular carcinoma in cirrhosis: a multivariate analysis of prognostic factors of tumor growth rate and patient survival. Hepatology 1992;16:132–7. 1352268 10.1002/hep.1840160122

[9] Sheu JC, Sung JL, Chen DS, Growth rate of asymptomatic hepatocellular carcinoma and its clinical implications. Gastroenterology 1985;89:259–66. 2408960 10.1016/0016-5085(85)90324-5

[10] Ebara M, Hatano R, Fukuda H, Natural course of small hepatocellular carcinoma with underlying cirrhosis. A study of 30 patients. Hepatogastroenterology 1998;45 Suppl 3:1214–20. 9730377

[11] Singal AG, Pillai A, Tiro J. Early detection, curative treatment, and survival rates for hepatocellular carcinoma surveillance inpatients with cirrhosis: a meta-analysis. PLoS Med 2014;ll:el001624. 10.1371/journal.pmed.1001624 PMC3972088 24691105

[12] van Meer S, de Man RA, Coenraad MJ, Surveillance for hepatocellular carcinoma is associated with increased survival: results from a large cohort in the Netherlands. J Hepatol 2015;63:1156–63. 26100498 10.1016/j.jhep.2015.06.012

[13] Rich NE, John BV, Parikh ND, Hepatocellular carcinoma demonstrates heterogeneous growth patterns in a multicenter cohort of patients with cirrhosis. Hepatology 2020;72:1654–65. 32017165 10.1002/hep.31159 PMC7398837

[14] Tong MJ, Kaki DA, Huynh CT, Raman SS, Lu DS. Tumor growth rates and recurrence-free survival in chronic viral hepatitis patients with hepatocellular carcinoma Hepatoma Res 2019;5:36.

[15] Brunt EM, Tiniakos DG. Histopathology of nonalcoholic fatty liver disease. World J Gastroenterol 2010;16:5286–96. 21072891 10.3748/wjg.v16.i42.5286 PMC2980677

[16] Tong MJ, Rosinski AA, Huynh CT, Raman SS, Lu DSK. Long-term survival after surveillance and treatment in patients with chronic viral hepatitis and hepatocellular carcinoma. Hepatol Commun 2017;1:595–608. 29404481 10.1002/hep4.1047 PMC5721434

[17] Kirn TK, Lee KH, Jang HJ, Analysis of gadobenate dimeglumine-enhanced MR findings for characterizing small (1–2-crn) hepatic nodules inpatients at high risk for hepatocellular carcinoma. Radiology 2011;259:730–8. 21364083 10.1148/radiol.11101549

[18] Lemon SC, Roy J, Clark MA, Friedmann PD, Rakowski W. Classification and regression tree analysis in public health: methodological review and comparison with logistic regression. Ann Behav Med 2003;26:172–81. 14644693 10.1207/S15324796ABM2603_02

[19] Friedman PJ. Classification of leiornyornatous lung Igfions. AJR Am Roentgenol 1984;142:851–2. 10.2214/ajr.142.4.851 6608255

[20] Grohmann M, Wiede F, Dodd GT, Obesity drives STAT-1-dependent NASH and STAT-3-dependent HCC. Cell 2018;175:1289 306.e20. 30454647 10.1016/j.cell.2018.09.053 PMC6242467

[21] Huang DQ, El-Serag HB, Loomba R. Global epidemiology of NAFLD-related HCC: trends, predictions, risk factors and prevention. Nat Rev Gastroenterol Hepatol 2021;18:223–38. 33349658 10.1038/s41575-020-00381-6 PMC8016738

[22] Noureddin M, Jones C, Alkhouri N, Gomez EV, Dieterich DT, Rinella ME; NASHNET. screening for nonalcoholic fatty liver disease in persons with Type 2 diabetes in the United States is cost-effective: a comprehensive cost-utility analysis. Gastroenterology 2020;159:1985–7.e4. 32763241 10.1053/j.gastro.2020.07.050

[23] An C, Choi YA, Choi D, Growth rate of early-stage hepatocellular carcinoma in patients with chronic liver disease. Clin Mol Hepatol 2015;21:279–86. 26523271 10.3350/cmh.2015.21.3.279 PMC4612289

[24] Nathani P, Gopal P, Rich N, Hepatocellular carcinoma tumour volume doubling time: a systematic review and meta-analysis. Gut 2021;70:401–7. 32398224 10.1136/gutjnl-2020-321040 PMC7657990

[25] Ioannou GN, Beste LA, Green PK, Increased risk for hepatocellular carcinoma persists up to 10 years after HCV eradication in patients with baseline cirrhosis or high FIB-4 scores. Gastroenterology 2019;157:1264–78.e4. 31356807 10.1053/j.gastro.2019.07.033 PMC6815714

[26] Benhammou JN, Moon AM, Pisegna JR, Nonalcoholic fatty liver disease risk factors affect liver-related outcomes after direct-acting antiviral treatment for hepatitis C. Dig Dis Sci 2021;66:2394–406. 32654086 10.1007/s10620-020-06457-2 PMC7854862

[27] Mehta N, Heimbach J, Harnois DM, Validation of a risk estimation of tumor recurrence after transplant (RETREAT) score for hepatocellular carcinoma recurrence after liver transplant. JAMA Oncol 2017;3:493–500. 27838698 10.1001/jamaoncol.2016.5116 PMC5395317

[28] Nault JC, Villanueva A. Biomarkers for hepatobiliary cancers. Hepatology 2021;73 Suppl 1:115–27. 32045030 10.1002/hep.31175

[29] Parikh ND, Singal AG. Model for end-stage liver disease exception points for treatment-responsive hepatocellular carcinoma. Clin Liver Dis (Hoboken) 2016;7:97–100. 31041039 10.1002/cld.545 PMC6490268

[30] Mehta N, Heimbach J, Lee D, Wait time of less than 6 and greater than 18 months predicts hepatocellular carcinoma recurrence after liver transplantation: proposing a wait time "sweet spot". Transplantation 2017;101:2071–8. 28353492 10.1097/TP.0000000000001752 PMC5568965

[31] Goldberg DS, Taddei TH, Serper M, Identifying barriers to hepatocellular carcinoma surveillance in a national sample of patients with cirrhosis. Hepatology 2017;65:864–74. 27531119 10.1002/hep.28765

[32] Aby ES, Winters AC, Lin J, A telephone and mail outreach program successfully increases uptake of hepatocellular carcinoma surveillance. Hepatol Commun 2020;4:825–33. 32490319 10.1002/hep4.1511 PMC7262281

[33] Simon TG, Duberg AS, Aleman S, Lipophilic statins and risk for hepatocellular carcinoma and death in patients with chronic viral hepatitis: results from a nationwide swedish population. Ann Intern Med 2019;171:318–27. 31426090 10.7326/M18-2753 PMC8246628

[34] Simon TG, Ma Y, Ludvigsson JF, Association between aspirin use and risk of hepatocellular carcinoma. JAMA Oncol 2018;4:1683–90. 30286235 10.1001/jamaoncol.2018.4154 PMC6440745

