# Supplementary material for: Nonalcoholic fatty liver disease-related hepatocellular carcinoma growth rates and their clinical outcomes
Source: Hepatoma Res. Author manuscript; Available in PMC 2021 Dec 28. (PMC8713558; doi:10.20517/2394-5079.2021.74)
Supplement: Supplemental figure 1 [file NIHMS1760040-supplement-Supplemental_figure_1.pptx]

## Slide 1
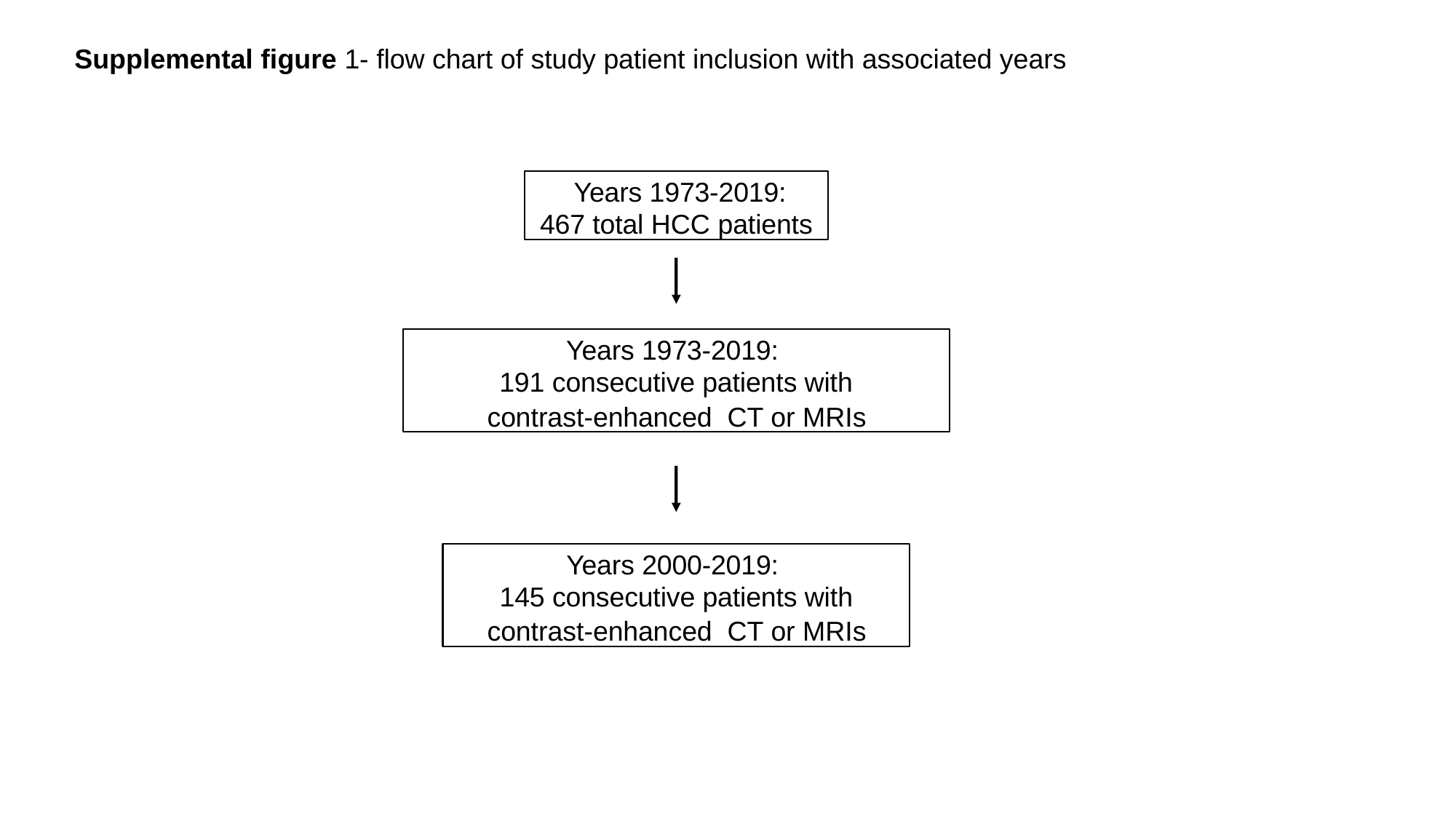

Supplemental figure 1- flow chart of study patient inclusion with associated years
 Years 1973-2019:
467 total HCC patients
Years 1973-2019:
191 consecutive patients with
contrast-enhanced CT or MRIs
Years 2000-2019:
145 consecutive patients with
contrast-enhanced CT or MRIs
