# Supplementary material for: Nonalcoholic fatty liver disease-related hepatocellular carcinoma growth rates and their clinical outcomes
Source: Hepatoma Res. Author manuscript; Available in PMC 2021 Dec 28. (PMC8713558; doi:10.20517/2394-5079.2021.74)
Supplement: Supplemental tables 1-3 [file NIHMS1760040-supplement-Supplemental_tables_1-3.docx]

**Supplemental Table 1-** Comparison of baseline characteristics between men and women in the NAFLD sub-cohort (n=47).

| **Baseline characteristic** | **Men (n=19)** | **Women (n=28)** | ***P* value** |
| --- | --- | --- | --- |
| ***Clinical*** | | | |
| Mean age at HCC dx ± SD | 66.0 ± 5.3 | 62.7 ± 7.6 | 0.144 |
| Median INR (IQR) | 1.2 (1.1-1.3) | 1.1 (1.1-1.3) | 0.972 |
| Median AST (IQR) | 37.5 (28-55) | 50.0 (39-65) | 0.071 |
| Median ALT (IQR) | 32.0 (23-39) | 31.0 (24-38) | 0.858 |
| Median albumin (IQR) | 3.6 (3.3-4.4) | 3.6 (3.1-3.9) | 0.408 |
| Median total bilirubin (IQR) | 1.4 (0.9-2.3) | 1.1 (0.7-2.4) | 0.354 |
| Median platelets (IQR) | 94.0 (54-147) | 98.5 (71-141) | 0.965 |
| Diabetes | 13 (68.4) | 22 (78.6) | 0.434 |
| Encephalopathy | 5 (26.3) | 6 (21.4) | 0.698 |
| Ascites | 7 (36.8) | 11 (39.3) | 0.866 |
| Family history HCC | 0 (0) | 2 (7.1) | 0.234 |
| Family history LD | 4 (21.1) | 12 (42.9) | 0.122 |
| Child-Pugh Score A | 8 (42.1) | 15 (60.0) | 0.071 |
| Child-Pugh Score B | 11 (57.9) | 7 (28.0) |  |
| Child-Pugh Score C | 0 (0) | 3 (12.0) |  |
| ***HCC*** | | | |
| Surveillance, n (%) | 14 (73.7) | 14 (50) | 0.104 |
| OLT | 8 (42.1) | 12 (42.9) | 0.889 |
| Resection | 0 (0) | 1 (3.6) |  |
| RFA | 6 (31.6) | 8 (28.6) |  |
| TACE | 1 (5.3) | 2 (7.1) |  |
| Chemotherapy | 2 (10.5) | 1 (3.6) |  |
| Supportive care | 2 (10.5) | 3 (10.7) |  |
| Median initial tumor size (IQR) | 2.3 (1.7-3.3) | 2.4 (1.8-2.9) | 0.854 |
| Within Milan, n (%) | 15 (79) | 27 (96.4) | 0.057 |

HCC: hepatocellular carcinoma; Dx: diagnosis; LD: liver disease; OLT= orthotopic liver transplantation; RFA: radiofrequency ablation; TACE: trans-arterial chemoembolization

**Supplemental table 2**- Comparisons of TGR according to the initial tumor size stratified by the etiology of HCC (n=145).

| **Tumor size (cm)** | **N** | **Median % TGR** | **IQR** | ***P* value** |
| --- | --- | --- | --- | --- |
| ***HBV*** | | | | |
| Unknown | 1 | -- | -- | 0.390 |
| 1-3 | 23 | 8.4 | 1.7-16.4 |  |
| 3-5 | 9 | 6.1 | 3.8-13.7 |  |
| 5-10 | 3 | 2.7 | -0.2-8 |  |
| >10 | 2 | 2.6 | 2.6-3 |  |
| ***HCV*** | | | | |
| Unknown | 1 | -- | -- |  |
| 1-3 | 39 | 6.1 | 2.5-12.3 | 0.368 |
| 3-5 | 16 | 3.4 | 0.5-7.5 |  |
| 5-10 | 5 | 8 | 0.3-16.2 |  |
| >10 | None | None | -- |  |
| ***NAFLD*** | | | | |
| Unknown | 1 | -- | -- | 0.369 |
| 1-3 | 3 | 3.8 | 1.5-11.3 |  |
| 3-5 | 9 | 5.4 | -2.2-14.6 |  |
| 5-10 | 3 | 3.4 | 0.8-5.6 |  |
| >10 | 1 | -10.1 | -- |  |

**Supplemental table 3**- Hazard ratios of overall mortality based on TGR growth rate by etiology and treatment modality in 145 patients from 2000-2019.

| **Hazard Ratios of Mortality according to TGR categories based on the tree model by Etiology** | | | | | | |
| --- | --- | --- | --- | --- | --- | --- |
| *Etiology* | *node* | *TGR* | *n* | *HR* | *95% CI* | *p-value* |
| NALFD | 1+2 | low | 33 | 1.000 | 1.000 | -- |
| NAFLD | 3+4 | medium | 10 | 0.368 | 0.047-2.910 | 0.343 |
| NAFLD | 5 | fast | 4 | 3.559 | 0.953-13.283 | 0.059 |
|  |  |  |  |  |  |  |
| Hep B + C | 1+2 | low | 65 | 1.000 | 1.000 | -- |
| Hep B + C | 3+4 | medium | 28 | 1.427 | 0.800-2.544 | 0.228 |
| Hep B + C | 5 | fast | 5 | 7.108 | 2.634-19.180 | 0.000 |
|  |  |  |  |  |  |  |
| **Hazard Ratios of Mortality according to TGR categories based on the tree model by Treatment** | | | | | | |
| *Treatment* | *node* | *TGR* | *n* | *HR* | *95% CI* | *p-value* |
| OLT | 1+2 | low | 24 | 1.000 | 1.000 | -- |
| OLT | 3+4 | medium | 12 | 0.334 | 0.040-2.779 | 0.310 |
| OLT | 5 | fast | 3 | 4.931 | 0.953-25.521 | 0.057 |
|  |  |  |  |  |  |  |
| RFA, Resection, TACE | 1+2 | low | 56 | 1.000 | 1.000 | -- |
| RFA, Resection, TACE | 3+4 | medium | 16 | 2.740 | 1.323-5.676 | 0.007 |
| RFA, Resection, TACE | 5 | fast | 3 | 7.408 | 2.091-26.246 | 0.002 |
